# Culture Shapes How We Look at Faces

**DOI:** 10.1371/journal.pone.0003022

**Published:** 2008-08-20

**Authors:** Caroline Blais, Rachael E. Jack, Christoph Scheepers, Daniel Fiset, Roberto Caldara

**Affiliations:** 1 Department of Psychology, University of Glasgow, Glasgow, United Kingdom; 2 Département de Psychologie, Université de Montréal, Montréal, Canada; University of Sydney, Australia

## Abstract

**Background:**

Face processing, amongst many basic visual skills, is thought to be invariant across all humans. From as early as 1965, studies of eye movements have consistently revealed a systematic triangular sequence of fixations over the eyes and the mouth, suggesting that faces elicit a universal, biologically-determined information extraction pattern.

**Methodology/Principal Findings:**

Here we monitored the eye movements of Western Caucasian and East Asian observers while they learned, recognized, and categorized by race Western Caucasian and East Asian faces. Western Caucasian observers reproduced a scattered triangular pattern of fixations for faces of both races and across tasks. Contrary to intuition, East Asian observers focused more on the central region of the face.

**Conclusions/Significance:**

These results demonstrate that face processing can no longer be considered as arising from a universal series of perceptual events. The strategy employed to extract visual information from faces differs across cultures.

## Introduction

It is a widely held belief that many basic visual processes are common to all humans, independent of culture. Face recognition is considered to be one such process, as this basic biological skill is necessary for effective social interactions. Any approach aiming to understand face perception must recognize, however, that only a small part of the visual information available on faces is actually used. Since the seminal work of Yarbus [1], we have known that humans use a series of foveal fixations to extract visual information to process faces, and that these sequences of eye fixations describe the way in which overt visual attention is directed [2]. Studies of eye movements have persistently revealed a systematic triangular sequence of fixations over the eye and the mouth, with dominance to the eyes e.g., [3], [4]–[7], suggesting that the presence of a face triggers a universal, biologically-determined information extraction pattern. However, this literature is based on observations with adults from Western cultures only. Consequently, the universality of these findings remains uncertain.

In the past decade, systematic differences between Westerners and East Asians have been found in a variety of perceptual tasks and paradigms for a recent review see [8]. Kitayama et al. [9] presented Western Caucasian and East Asian observers with a square containing a line. Observers were then presented with squares of various sizes and asked to draw a line that was identical to the first line in either *absolute* or *relative* length to the previously seen surrounding square. Western Caucasian observers were more accurate in *absolute* judgments, whereas East Asian observers were more accurate in the *relative* task. These observations suggest that Westerners have *analytic* strategies relating to focal information (i.e. the line only), whereas East Asians have optimal *holistic* strategies for encoding contextual information (i.e. the line within the square). Note that the term holistic here refers to the definition used in the cultural framework and does not refer to the term and mechanisms relating to the holistic processing used in the face perception literature. We will use the term *holistic* in its cultural context throughout the paper.

Convergent evidence supporting cultural diversity has been found in scene perception [10], [11], description [10] and categorization [12], showing that Westerners focus analytically on salient objects and use categorical rules when organizing their environment. By contrast, people from China, Korea and Japan - all East Asian cultures - focus more holistically on relationships and similarities among objects when organizing the environment. All these studies point to a similar pattern of results, revealing that orthogonal mechanisms influence visual perception and categorization across cultures.

Until recently it was unknown whether these cultural perceptual differences arise at the encoding, retrieval or more elaborate stages of information processing. Tracking eye movements is an appropriate approach to address this issue, as sequences of eye fixations describe the way in which overt visual attention is directed and the intake of information is isolated [2]. Indeed, recent eye-tracking data provided direct evidence that cultural backgrounds shape visual environment affordance. Western American observers had longer fixations on focal objects during scene processing compared to Chinese observers, whereas Chinese observers fixated more on the background compared to Western American observers [13]. Cultural perceptual biases therefore seem to arise from differences in what observers attend to in a scene, and what information is extracted to achieve perception.

Previous literature on culture has focused on visual scene processing or simple visual categorization tasks. Natural scenes are heterogeneous visual inputs with complex statistical properties [14]. However, human faces are homogenous objects, roughly symmetrical, that share similar salient shapes arranged in fixed locations across exemplars (e.g. two eyes above a central nose and mouth). It is unclear whether these perceptual cultural differences in scene processing would generalize to the biologically relevant class of human faces, since faces are arguably the most important and salient visual stimulus a human ever encounters. The ability to identify conspecifics from the face is of primary interest for human social behavior and is routinely and effortlessly achieved in every culture. For this reason, perhaps, literature on face processing, and in particular studies of eye movements, has so far largely ignored the role of culture and generalized findings of Western Caucasian observers to the entire human population.

Human beings have developed through social experience a natural expertise at extracting information from faces (identity, race, gender, age, emotional state, etc.), with the exception of other-race/ethnic group face recognition [15]; a well-known phenomenon often reported in the literature as the other-race effect [16]. Despite numerous studies having investigated the other-race effect for more than thirty years for a review see [17], it is primarily still unknown (i) whether people from different cultures process faces using the same perceptual strategies and (ii) whether they adapt visual information extraction as a function of the race of the input face.

To address these issues, we monitored the eye movements of fourteen Western Caucasian and fourteen East Asian observers during the learning and recognition stages of a face recognition task and a subsequent face categorization by race task, using Western Caucasian and East Asian faces. The expression of the familiar faces was changed between learning and recognition to prevent trivial image matching strategies to memorize face identities. Also, to prevent anticipatory strategies and to ensure that the location of the first fixation on the faces is self-determined by the observer, faces were pseudorandomly presented in one of four quadrants of a computer screen. To recognize faces, observers must identify *unique* sets of facial features - a taxing constraint on information extraction, which might modulate facial scanpaths. To control for this factor, we subsequently recorded eye movements of the same observers in the less demanding task of face categorization by race [18], which relies on using information *common* to faces of the same race [19]. We observed a striking cultural contrast across tasks between the fixation scanpaths of our two cultural populations: Western Caucasian observers consistently fixated the eye region, and partially the mouth, whereas East Asian observers fixated more on the central region of the face.

## Results

### Behavioral

#### Face recognition

**Figure 1:**
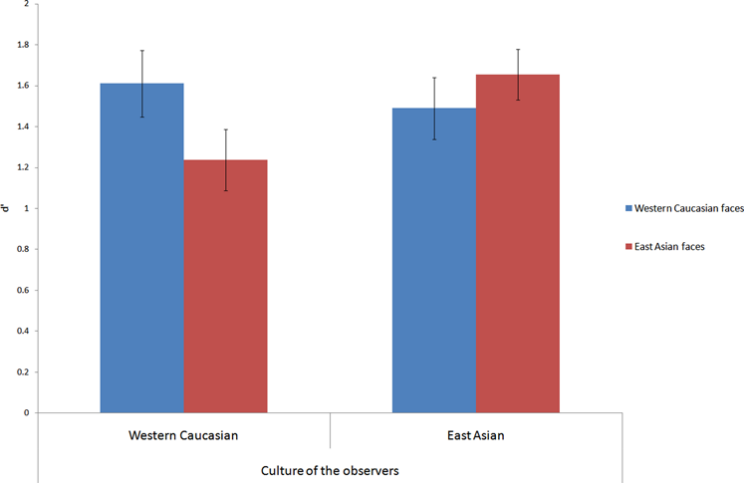
*d′* accuracy scores of the old/new face recognition paradigm, for Western Caucasian and East Asian observers. Error bars report standard errors of the mean. Observers recognized same-race faces significantly better than other-race faces.

A two-way mixed design ANOVA including *Race of the face* and *Culture of the observer* as respectively within- and between-subjects variables revealed a significant interaction: observers of both cultures were more accurate (*d′*) in judging familiarity for same- than other-race faces (F(1, 26) = 12.86, *p* = .001, η^2^ = 0.330) (Figure 1).

*Post hoc* two-tailed paired *t-tests* revealed that the advantage in recognizing same-race faces was present in both groups of observers (Western Caucasians (*p*<.01); East Asians (*p*<.04)). Neither the main effect of *Race of the face* (F(1, 26) = 1.93, *p* = .17, η^2^ = .069) nor the *Culture of the observer* (F(1, 26) = .59, *p* = .44, η^2^ = .022) reached significance.

As for correct response times (Table 1), neither the main effect of *Race of the face* (F(1, 26) = 2.96, *p* = .10, η^2^ = .102) nor *Culture of the observer* (F(1, 26) = 1.04, *p* = .31, η^2^ = .038) reached significance. The interaction between these factors also failed to reach significance (F(1, 26) = 2.36, *p* = .13, η^2^ = .083).

**Table 1:** Response times (RT) and number of fixations for Western Caucasian (WC) and East Asian (EA) observers (obs) during WC and EA face learning, recognition and categorization by race.

	Learning				Recognition				Classification			
	WC obs		EA obs		WC obs		EA obs		WC obs		EA obs	
	WC faces	EA faces	WC faces	EA faces	WC faces	EA faces	WC faces	EA faces	WC faces	EA faces	WC faces	EA faces
*RT*	—	—	—	—	1567 (122)	1723 (134)	1478 (112)	1486 (100)	751 (50)	764 (52)	692 (33)	701 (27)
*Fixations*	14.2 (0.6)	14.2 (0.6)	14.5 (0.5)	14.4 (0.6)	4.8 (0.4)	5.3 (0.5)	4.4 (0.4)	4.5 (0.3)	2.2 (0.2)	2.3 (0.2)	2 (0.1)	2.1 (0.1)

Numbers in parenthesis report the (±) standard errors of the mean. Note that the presentation time was fixed during learning (5 seconds).

#### Categorization by race

Observers of both cultures showed ceiling effects for accuracy (98% of correct answers). A two-way mixed design ANOVA with *Race of the face* as within-subjects factor and *Culture of observer* as between-subjects factor did not reveal any significant differences for response times (Table 1). Neither the main effect of *Race of the face* (F(1, 26) = .78, *p* = .38η^2^ = .029), nor *Culture of the observer* (F(1, 26) = 1.11, *p* = .30, η^2^ = .041) reached significance. The interaction between these factors also failed to reach significance, (F(1, 26) = .021, *p* = .88. η^2^ = .0008).

### Eye movements

On average, observers performed about 14 fixations per trial during learning and 5 fixations during recognition (Table 1). Note that faces were pseudorandomly presented in one of four quadrants of a computer screen for 5 s during learning and until response during recognition (*M* = 1563 ms) and categorization (*M* = 726 ms).

**Figure 2:**
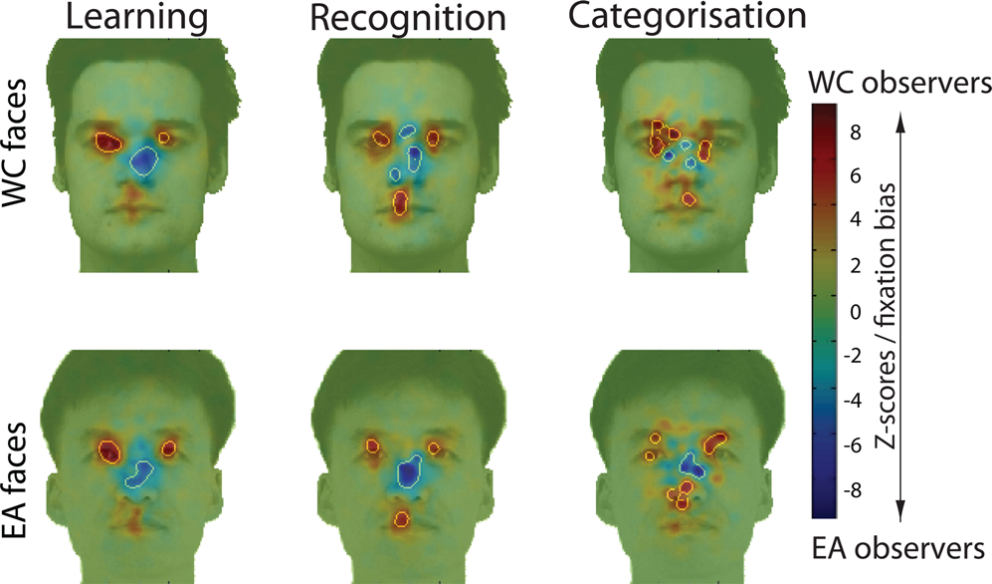
Fixation biases for Western Caucasian (WC - red) and East Asian (EA - blue) observers are highlighted by subtracting WC and the EA Z-scored fixation distribution maps during WC and EA face learning, recognition and categorization by race. Areas showing a significant fixation bias are delimited by white borders (Z*_crit_*>|4.25|; *p*<.05); values near 0 indicate similar magnitude in fixation between observers from different cultures.

Western Caucasian and East Asian observers differed in how they extracted facial information using eye movements: Figure 2 shows significant differences in fixation locations, as revealed by a two-tailed *Pixel test* [20] (Z*_crit_*>|4.25|, *p*<.05 – see Methods and Figure S1 for details).

**Figure 3:**
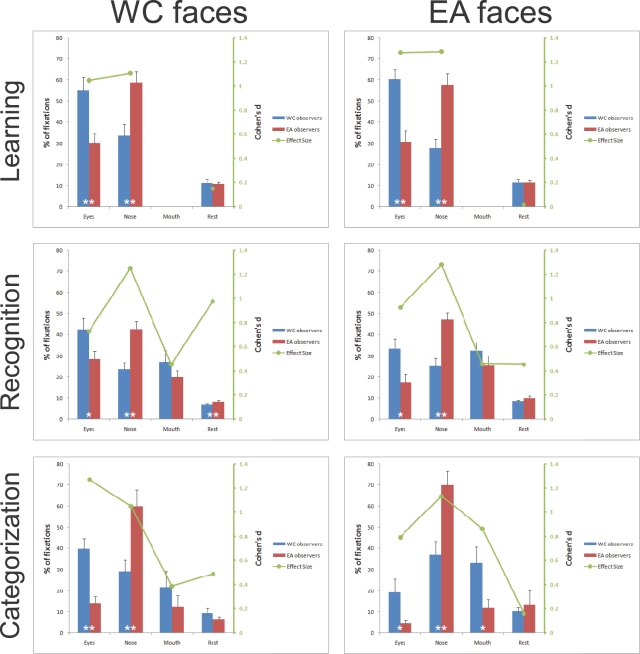
Left *y* axis: Percentages of fixations landing in the eye, nose, mouth and rest of the facial regions across tasks (WC = Western Caucasian; EA = East Asian). Error bars report standard errors of the mean. Significant differences between observers from different cultures are reported at the bottom of the bars (* = *p*<.05; ** = *p*<.01). Right *y* axis: *Cohen's d* (1988) effect size values. Note, that the absence of fixations for the mouth region in the learning condition is due to the lack of significant fixation biases in this region as defined by the fixation map analysis (see the Methods section for details).

To further investigate the magnitude of the fixation biases across cultures, we calculated the percentage of fixations landing within these face regions and the rest of the face. Two-way mixed design ANOVAs with *Face regions* as within-subject factor and *Culture of the observer* as a between-subjects factor revealed significant interactions for those factors in all conditions (*p*<.001 - Fig. 3).

In general, Western Caucasian observers had significantly more fixations landing in the eye region, while East Asian observers had more fixations on the nose region, as revealed by independent two-tailed *t-tests* carried out on the percentage of fixations in these regions for each task separately (Fig. 3). Both cultural biases for these facial features were reliable and robust, as highlighted by the large magnitude of *Cohen's d* effect size values.

Notably, observers from a given culture fixated the same face regions regardless of the race of the face. Western Caucasian observers prominently fixated the eye region during learning and recognition. In contrast, East Asians consistently fixated the central region of the face. The less demanding race categorization task elicited eye movement patterns similar to the more taxing face recognition, even with only 2 fixations on average (Table 1). Statistical analyses on the number of fixations did not reveal any significant effect, with the exception of a marginal main effect of smaller number of fixations for Western Caucasian faces (*M* = 4.56) compared to East Asian faces (*M* = 4.89) during face recognition (F(1, 26) = 4.63, *p* = .04, η^2^ = .151).

**Figure 4:**
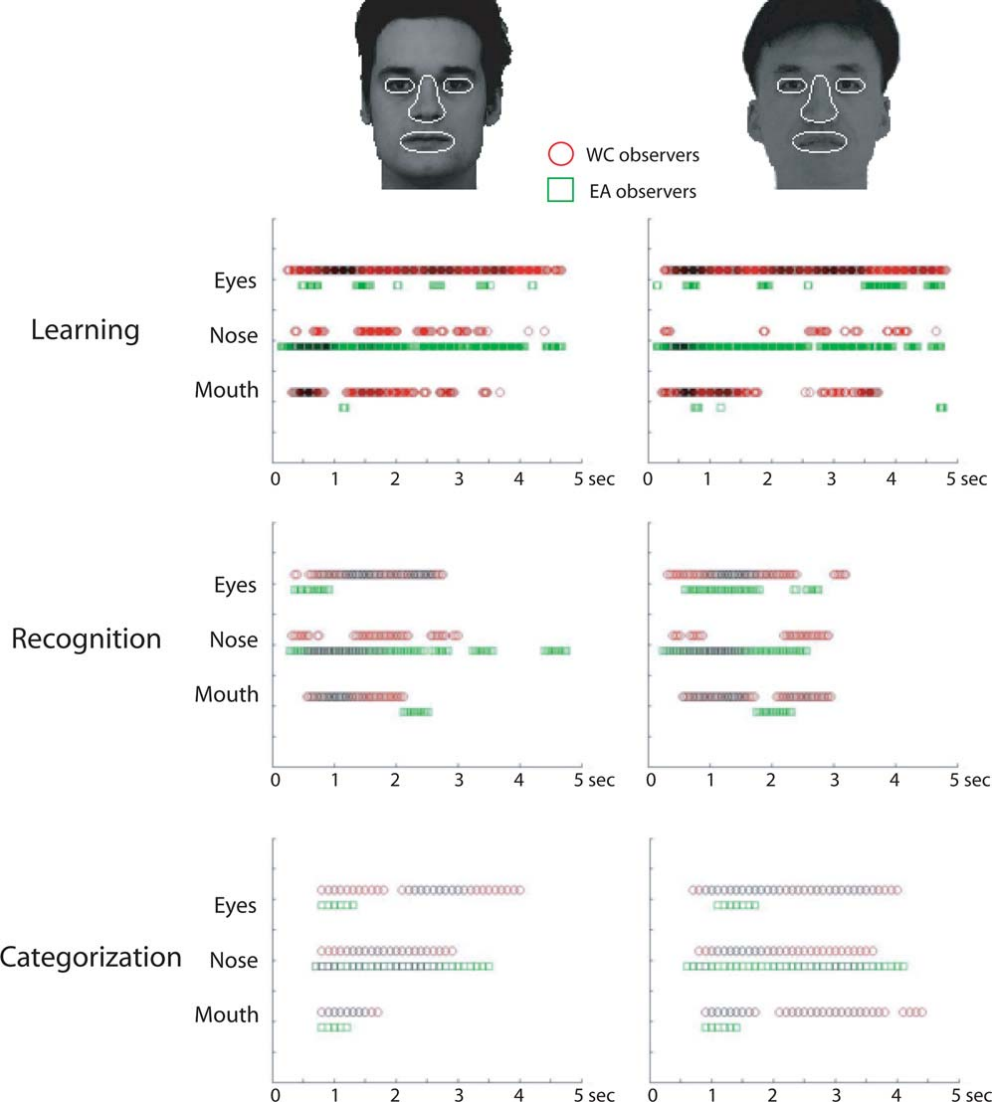
Time course of significant fixations on facial features (eyes, nose and mouth) during face learning, recognition and categorization by race, for WC (red square - top rows) and EA (green circle - bottom rows) observers (Z*crit*>4.64; *p*<.05). The significant facial feature fixations are weighted by the surface area they covered within the region of interest; with darker outlines relating to wider regions.

The time course of fixation maps during learning, recognition and categorization by race was tracked by computing fixation maps on 20 ms time slots separately for each group of observers and applying a one-tailed *Pixel test* (Z*_crit_*>4.64; *p*<.05 – see Methods for details). The time slots in which fixations landing on the eye, nose and mouth regions reached significance are reported in Figure 4.

This analysis highlights the consistency of the bias towards the eyes (and partially the mouth) by Western Caucasian observers and the nose, the central region of the face, by East Asian observers across time and tasks (see also the supporting QuickTime™ movies for these tasks: Video S1: learning, Video S2: recognition and Video S3: categorization).

## Discussion

We report a striking cultural contrast: Western Caucasian observers consistently fixated the eye region, and partially the mouth, whereas East Asian observers fixated more on the central region of the face to extract information from faces. This finding was consistent when fixation scanpaths were compared across three different face processing tasks: learning, recognition and categorization by race.

These results demonstrate that people from different cultures achieve human face processing by focusing on different face information. Direct or excessive eye contact may be considered rude in East Asian cultures [21] and this social norm might have determined gaze avoidance in East Asian observers. To some extent our findings are consistent with observations that Westerners tend to engage analytic perceptual strategies for processing the visual environment, whereas East Asians use holistic perceptual strategies [8], [9], [11]–[13]. Westerners might allocate their attention to single facial features (i.e. eyes and the mouth) with a bias towards the eyes to effectively learn and recognize faces. This triangular scan pattern strategy is fully consistent with previous eye movements findings [1], [4]–[7], [22], [23] and also in line with recent neuroimaging results on Western Caucasian observers showing a tuning in the neural face-sensitive regions for visual stimuli containing more elements in the upper part [24]. East Asians on the other hand would recognize faces by focusing in the region that would be optimal and economical to integrate information holistically: the center of the face (i.e. nose). Because retinal cell density and visual resolution decrease steeply towards the peripheral visual field, the center of the face is likely to be the most advantageous spatial position to capture facial feature information globally. Both strategies used by East Asian observers (social norm and holistic/global processing) could account for the East Asian fixation bias towards the nose region. The present data do not allow us to disentangle whether only one or both of these explanations are valid and future studies are necessary to directly address this issue. Nevertheless, both explanations reflect mechanisms shaped by culture and do not alter our main conclusions. In addition, it is worth noting that to the best of our knowledge these strategies cannot be explained by the differences in faces *per se*. Both objective anthropometric measures [25], [26] and computational models [19] have revealed comparable heterogeneity for Caucasian, African and East Asian faces.

These findings are not a straightforward extension of previous eye tracking results recorded during scene perception [13]. While exploring visual scenes, East Asian observers made more (scattered) fixations to the background compared to Western Caucasian observers, which instead focused more on central objects present in the scene. The present findings on face processing show the opposite pattern. East Asian observers focused on a focal region (i.e. the center of the face), whereas Western Caucasian observers sampled more largely the visual input space (i.e. scattered fixations across facial features). However, visual natural scenes are complex heterogeneous stimuli engaging wide saccadic eye movements. In our experiment, a single face was presented on a neutral background, constituting a salient narrow stimulation for the visual system. This discrepancy in eye movement patterns between faces and visual scenes highlights the specificity of the mechanisms involved to resolve the visual task (and categories) at hand. Nevertheless, importantly, although the precise nature of the visual strategies used across these tasks remains to be clarified, both findings show a marked cultural diversity in eye movements.

As expected, observers of both races were more accurate at recognizing same- than other-race faces, as found in many studies on the other-race effect [17]. Critically, however, previous studies on the other-race effect did not isolate the information on which the observers relied during face recognition and did not define the perceptual strategies they adopted. Therefore, the question of whether information used for face recognition is modulated by the race of the input faces is long standing within the cross-cultural face literature [17]. We reveal that Westerners focus on the eyes, whereas East Asians focus on the nose, regardless of the race of the faces and the task at hand. Observers do not change perceptual strategies as a function of the race of the face, highlighting the robustness of the perceptual mechanisms engaged during face processing. In addition, observers from different cultures reached a comparable behavioral performance by using different scanpaths, which, at first sight, could appear puzzling. However, eye movements do not provide direct evidence on the mental representations elaborated and used by the observers to solve the task. It remains possible that observers of both cultural groups constructed comparable representations for processing faces despite using different scanpaths. Future studies combining eye movements with a rigorous control of the presented information might help to clarify this issue.

Psychologists and philosophers have long assumed that while culture impacts on the way we think about the world, basic perceptual mechanisms are common among humans [27]. We provide evidence that social experience and cultural factors shape human eye movements for processing faces, which contradicts the view that face processing is universally achieved.

## Methods

### Participants

Fourteen Western Caucasian (6 males, 9 females) and 14 East Asian (7 males, 8 females) young adults (mean age 24.4 years and 23.2 years respectively) participated in this study. All East Asian participants were newly enrolled international students attending the University of Glasgow, having being born in East Asia and arriving in a Western country (Glasgow, UK) for the first time. The average duration of residence in the UK upon testing was 1 week within the East Asian group. The East Asian group comprised 8 Chinese and 6 Japanese students. All participants had normal or corrected vision and were paid £6 per hour for their participation. All the participants gave written informed consent and the protocol was approved by the departmental ethical committee.

### Materials

Stimuli were obtained from the KDEF [28] and AFID [29] databases and consisted of 56 East Asian and 56 Western Caucasian identities containing equal numbers of males and females. The images were 382×390 pixels in size, subtending 14° degrees of visual angle vertically and 10° degrees of visual angle horizontally. Images were viewed at a distance of 57 cm, which represents the size of a real face (approximately 20 cm in height) viewed from a distance of about 80 cm, reflecting a natural distance during human interaction. All the pictures were cropped around the face to remove clothing. Male faces were clean-shaven and none of the faces had particularly distinctive features (scarf, jewelry, etc.). Faces were aligned on the eye and mouth positions, their luminance was normalized and were presented on a 1024×768 pixel white background and displayed on a 21″ Iiyama HM204DTA monitor with a refresh rate of 120 Hz. Presentation of stimuli was controlled by the SR Research ExperimentBuilder software, version 1.4.202.

### Eye tracking

Eye movements were recorded at a sampling rate of 500 Hz with the EyeLink II head-mounted eye-tracker (SR International), which has an average gaze position error of <0.5°, a resolution of 1 arc min and a linear output over the range of the monitor used. Only the dominant eye of each participant was tracked although viewing was binocular. A manual calibration of eye fixations was conducted at the beginning of each task (and once every maximum 28 trials thereafter) using a nine-point fixation procedure as implemented in the EyeLink API software (see EyeLink Manual for details). The calibration was then validated with the EyeLink API software and repeated when necessary until the optimal calibration criterion was reached. At the beginning of each trial, participants were instructed to fixate a dot at the center of the screen to perform an automatic drift correction.

### Procedure

Observers were informed that they would be presented with a series of faces to learn and subsequently recognize, and that there would be two blocks of learning and recognition per race condition. In each block, observers were instructed to learn 14 face identities randomly displaying either neutral, happy or disgust expressions (7 females). After a 30 second pause, a series of 28 faces (14 faces from the learning phase – 14 new faces; 7 females) were presented and observers were instructed to indicate as quickly and as accurately as possible whether each face was familiar or not by pressing on a two button control pad using their dominant hand. Faces of the two races were presented in separate blocks, with the order of presentation for same- and other-race blocks being counterbalanced across observers. After the completion of the face recognition tasks, observers were instructed to perform a face categorization by race task on 56 Western Caucasian (28 women) and 56 East Asian (28 women) faces. Participants were required to indicate as quickly and as accurately as possible the race of the presented faces (‘Caucasian’ or ‘Asian’) by pressing on a two button control pad using their dominant hand. Response buttons were counterbalanced across participants for both tasks.

Each trial consisted of the presentation of a central fixation dot (which also served as an automatic drift correction) followed by a face presented pseudorandomly in one of four quadrants of the computer screen. Faces were presented for 5 seconds duration in the learning phase and until the observer responded in the recognition and race categorization phase. Each face was subsequently followed by the central fixation dot which preceded the next face stimulus.

### Data analyses

Only correct trials were analyzed. Fixation distribution maps were extracted individually for Western Caucasian and East Asian observers and face race, for the learning, recognition and categorization tasks separately. The fixation maps were computed by summing, across all (correct) trials, the fixation location coordinates (*x*, *y*) across time. Since more than one pixel is processed during a fixation, we smoothed the resulting fixation distributions with a Gaussian kernel with a sigma of 10 pixels (supporting Figure S1a). Fixation maps of all observers belonging to the same cultural group were then summed together separately for each face condition, resulting in *group* fixation maps (see supporting Figure S1a - top right for an example of such a map).

We then Z-scored the resulting group fixation maps by assuming identical Western Caucasian and East Asian eye movement distributions for a particular face race as the null hypothesis. Consequently, we pooled the fixation distributions of observers for both groups and used the mean and the standard deviation for Western Caucasian and East Asian faces to separately normalize the data (supporting Figure S1b). Finally, to clearly reveal the difference of fixation patterns across observers of different cultures, we subtracted the group fixation maps of the East Asian observers from the group Western Caucasian and we Z-scored the resulting distribution (supporting Figure S1c). To establish significance, we used a robust statistical approach correcting for multiple comparisons in the fixation map space, by applying a two-tailed *Pixel test* [20] on the differential fixation maps with the following threshold (*Z_crit_*>|4.25|; *p*<.05 – areas delimited with white borders in supporting Figure S1c). Thereafter, to further highlight the magnitude of the differences in fixation locations between observers from different cultures, we used the significant regions revealed by the *Pixel test* as a visual mask to define region of interest (e.g., roughly landing on the eyes, nose, and mouth; we also included the rest of the facial information in this analysis). We then calculated the percentage of fixations landing on those regions. To control for differences in size of the different regions of interest, we normalized the number of fixations by the area covered by the region of interest. Finally, *Cohen's d* effect size [30] was calculated within each region of interest, by pooling the standard deviations of both groups in the conditions of interest.

A similar procedure was applied to isolate the most fixated areas throughout the time course. That is, we constructed 3-D fixation maps (*x* coordinate, *y* coordinate and time) by dividing each trial into 20 ms bins, and calculated for each of the bins the number of times a given location (*x*, *y*) was fixated across all the trials. We then smoothed these 3-D fixation maps using a Gaussian kernel with a sigma of 10 pixels in the spatial domain and of 20 ms in the temporal domain. Finally, we transformed the 3D maps into Z-scores, by using the mean and standard deviation across all the dimensions and applied a one-tailed *Pixel test* to isolate the areas that elicited significant fixations (*Z_crit_*>4.64; *p*<.05). The statistical threshold provided by the *Pixel test* corrects for multiple comparisons across time and space while taking the spatial and temporal correlation inherent to eye movement data sampling into account [20]. We then defined three regions of interest: the eyes, the nose and the mouth (see Fig. 4) in order to visualize the significant fixation effects over time on the 3-D maps (see also the supporting QuickTime™ movies). We highlighted the presence of significant fixations in these regions by weighting the results as a function of the number of pixels activated. Note that Figure 4 relies on the *absolute* (see group fixation maps in the supporting Figure S1b for an example), rather than the *differential* fixation maps (Fig. 2).

## Supporting Information

Figure S1 Processing steps for the computation of fixation map biases for Western Caucasian (WC - *c*: red) and East Asian (EA - *c*: blue) observers during the face learning, recognition and categorization by race tasks. Please refer to the main text for details.
(15.14 MB DOC)

Video S1 Learning. Time course of the Z-scored fixation maps for Western Caucasian (WC) and East Asian (EA) observers during the learning stage of the face recognition task, using Western Caucasian and East Asian faces.
(7.07 MB MOV)

Video S2 Recognition. Time course of the Z-scored fixation maps for Western Caucasian (WC) and East Asian (EA) observers during the recognition stage of the face recognition task, using Western Caucasian and East Asian faces.
(1.27 MB MOV)

Video S3 Categorization. Time course of the Z-scored fixation maps for Western Caucasian (WC) and East Asian (EA) observers during the face categorization by race task, using Western Caucasian and East Asian faces. Note that on average observers used only 2 fixations to solve this task.
(1.27 MB MOV)
