# Supplementary figures and images for: Culture Shapes How We Look at Faces
Source: PLoS One. 2008 Aug 20;3(8):e3022. doi: 10.1371/journal.pone.0003022 (PMC2515341; doi:10.1371/journal.pone.0003022)

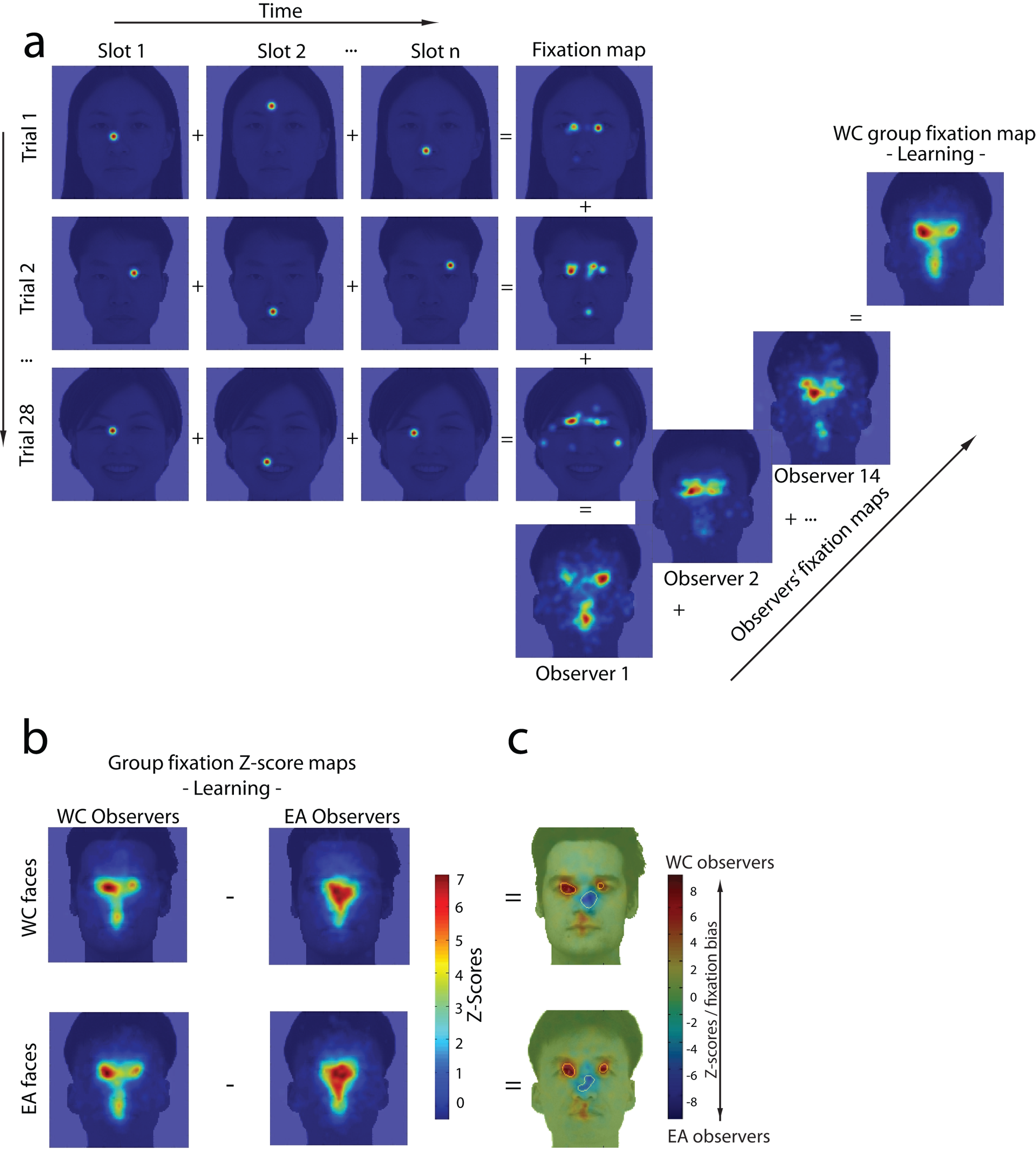

Supplement: Figure S1 — Processing steps for the computation of fixation map biases for Western Caucasian (WC - c: red) and East Asian (EA - c: blue) observers during the face learning, recognition and categorization by race tasks. Please refer to the main text for details. (15.14 MB DOC) [file pone.0003022.s001.tif]
